# Supplementary material for: Rutin-Activated Nuclear Factor Erythroid 2-Related Factor 2 (Nrf2) Attenuates Corneal and Heart Damage in Mice
Source: Pharmaceuticals (Basel). 2024 Nov 12;17(11):1523. doi: 10.3390/ph17111523 (PMC11597448; doi:10.3390/ph17111523)
Supplement: Supplementary file 1 [file pharmaceuticals-17-01523-s001.zip › pharmaceuticals-3258828-supplementary.pdf]

Supplementary Table S1. Interactions between rutin and amino acid residues of Keap (PDB ID: 6lrz).

Ligand	Pubchem ID	Binding energy	Ligand efficiency	Intermole energy	Ligand atoms (ring)	Docked amino acid residue (bond length)
					conventional hydrogen bond	
					C4″′-OH	Chain A: VAL\`608′O′ (1.95 Å)
					C3′′′-OH	Chain A: VAL\`369′O′ (1.84 Å)
					C4′′-OH	Chain A: THR\`560′OG1′ (2.16 Å)
					C3′′-OH	Chain A: ILE\`559′O′ (1.57 Å)
					C2′′-OH	Chain A: VAL\`606′O′ (2.26 Å)
					C3′-O (B)	Chain A: VAL\`514′HN′ (2.69 Å)
					C4′-OH (B)	Chain A: VAL\`467′O′ (2.89 Å)
					C4-O (C)	Chain A: GLY\`367′HN′ (2.45 Å)
					C7-OH (A)	Chain A: ALA\`510′O′ (2.12 Å)
Rutin	5280805	-8.69	-0.2	-12.08	Carbon–hydrogen bond	
					C3″-O	Chain A: THR\`560′CA′ (2.54 Å)
					C7-O (A)	Chain A: GLY\`464′CA′ (3.19 Å)
					Alkyl hydrophobic interaction	
					C6	Chain A: VAL\`608′O′ (3.86 Å)
					Pi-alkyl hydrophobic interaction	
					O	Chain A: ALA\`366′ (5.26 Å)
					O	Chain A: ALA\`366′ (5.29 Å)
					Miscellaneous bond (Pi–Sigma interaction)	
					O	Chain A: ALA\`466′CA′ (3.58 Å)

The molecular docking study shows nine conventional hydrogen bonds; two carbon–hydrogen bonds and a Pi-alkyl hydrophobic interaction; one alkyl hydrophobic interaction; and a Pi–Sigma interaction between rutin and the Keap (PDB ID: 6lrz) protein. C—carbon number; binding energy—kcal/mol; ligand efficiency—kcal/mol; Å—angstrom; VAL—valine; THR—threonine; ILE—isoleucine; GLY—glycine; ALA—alanine.
